# Red blood cell transfusion in a tertiary Haitian hospital’s emergency department: patient characteristics and availability challenges

**DOI:** 10.1186/s12245-024-00672-8

**Published:** 2024-09-27

**Authors:** Flawendjee Djaweelentz Jacques, Samentha Cyndie Julmisse, Ange Cindy Laurore, Ralph Mackenson Lefruit, Maurice Junior Chery, Kobel Dubique

**Affiliations:** Hôpital Universitaire de Mirebalais, Mirebalais, Center Haiti

**Keywords:** RBC transfusion, Transfusion practice, Emergency medicine, Haïti

## Abstract

**Background:**

Red blood cell (RBC) transfusions are essential for many patients admitted to emergency departments (ED). However, accessibility to red cell transfusions is often limited, especially in low-income countries such as Haiti. This article aims to determine the proportion of patients requiring emergency transfusion, transfused patients’ characteristics, as well as the response rate and timeliness of blood product delivery for requests made.

**Methods:**

A retrospective study was conducted among all patients with RBC transfusion indications from January to June 2022 at the ED of Mirebalais Teaching Hospital. The parameters studied included transfusion indications, pre-transfusion hemoglobin levels, and delay from prescription to transfusion.

**Results:**

During the study period, 3993 patients received treatment in the ED. The proportion of patients requiring RBCs was 7.69%, including 145 males and 117 females, with a median age of 43 [30–56] years. Only 21.7% of these patients received a transfusion. The average pre-transfusion hemoglobin level was 4.75 ± 1.68 g/dL. The most common transfusion’s indications were infection/sepsis (36.74%), trauma (23.48%), and cancer (21.57%). The median time delay from prescription to transfusion was 2.37 [0.97–4.93] days. The study identified significant associations between RBC transfusion probability and patient factors like hemoglobin levels, patient disposition, urgency of RBC request, and length of stay.

**Conclusion:**

RBC transfusion requests are frequent in the ED of Mirebalais Teaching Hospital, with a relatively high transfusion delay. Further studies on the relevance of RBC requests and ways to reduce delay from prescription to transfusion would be beneficial to improve this situation.

## Introduction

Blood transfusion is an essential treatment, crucial for patients with acute blood loss or severe anemia in healthcare systems [1]. However, blood shortages are a well-documented problem, especially in low- and middle-income countries, due to a lack of blood donors [1, 2] Low-income countries have a blood donation rate 5.0 per 1000 people compared to 31.5 in high-income countries [3]. Furthermore, the poorest countries, where 82% of the world’s population resides, donates only 39% of the global blood supply [4]. The frequent need for urgent life-saving transfusions further compounds the problem of blood product shortage [5]. This situation is often encountered in EDs where indications for blood product transfusions are diverse and numerous mainly for cancer, trauma, and active bleeding [6, 7].

Delayed blood transfusions may lead to adverse outcomes for patients [1]. In certain African countries, the average emergency department transfusion time can extend to six hours [8, 9].

The minimum recommended hemoglobin threshold for blood transfusions is typically 7 g/dL, although this value can be adapted based on factors such as symptoms, age, and underlying pathology. This threshold is frequently surpassed in patients with acute coronary syndrom, acute ischaemic stroke or subarachnoid haemorrhage [10, 11] Some observational studies have shown that pre-transfusion hemoglobin levels can range from 3.7 to 9.6 g/dL [9, 12,7] The rates of ED transfusions and demographic characteristics of those receiving transfusions can differ notably between countries [9, 8, 13].

In this context of challenges related to access to blood products, particularly in low-income countries, this article focuses on the specific situation in Haiti. It aims to determine the proportion of patients requiring emergency transfusion, transfused patients’ characteristics, as well as the response rate and timeliness of blood product delivery for requests made in the emergency department of a tertiary Haitian hospital. The study’s findings will be crucial for a better understanding of thе blood transfusion rеquirеmеnts in this hospital’s еmеrgеncy department. To the best of our knowledge, this is the first study of its kind to be conducted in Haiti, and it could serve as a foundation for further research aimed at enhancing the efficiency and relevance of blood transfusions in the emergency departments of low-income countries, where blood product availability remains a significant issue.

## Materials and methods

### Study design

This was a retrospective study conducted in a Haitian tertiary hospital.

### Study site

The study was carried out over a six-month period in the ED of the Mirebalais Teaching Hospital, located in a suburban area of the country, serving the population of the ten departments. The hospital was built by Zanmi Lasante /Partners in Health, a health and social justice organization in partnership with the Haitian Ministry Of Health, and it has an annual census of more than 14,000 patients.

### Population (selection, recruitment, and enrollment)

The target population included all patients attending the ED of Mirebalais Teaching Hospital from January to June 2022. All patients requiring RBC transfusion during their ED visit, identified through a request made by the treating physician, were eligible for inclusion, regardless of age, for traumatic cases. For non-traumatic conditions, only patients aged ≥ 16 years were included. This was because pediatric patients (< 16 years) with non-traumatic conditions were managed directly by the pediatrics department. Patients were excluded if they left the ED before the request or had illegible or incomplete request forms (absence of an electronic medical record (EMR) code, date, and time of request).

The RBC request process begins with the completion of the form by the physician. This form is accompanied by a blood sample taken by the nurse and then transported to the blood bank by an available nurse’s aide or intern. In cases of emergency, the blood bank is contacted directly by phone or the physician goes there personally. Although a protocol for massive transfusion exists theoretically at the hospital, the shortage of RBCs, platelets, or fresh frozen plasma renders this practice impossible, even if a situation requiring it is recognized in the ED. For the same reason, the blood bank rarely provides the exact quantity of requested RBCs. For example, a patient who needs three packs of blood may receive them staggered, one pack at a time, with long intervals between each transfusion.

Once the request for RBCs was made, if the patient was transfused but need more transfusion, the old request was verbally reactivated at the blood bank, thus avoiding the creation of a new request. However, a request may be issued twice if the initially responsible physician did not adequately transmit information to the next on-duty emergency physician. Therefore, duplicate occurrences were eliminated. Thus, a request form was associated with each medical record and each unique patient.

### Instrument and variables

A structured data collection form was developed to capture variables from patient charts and RBC request forms, including demographic data (age, sex, geographic origin), clinical details (pre-transfusion hemoglobin level, transfusion indication), request details (number of RBC units requested, urgency level of RBS request), transfusion details (number of RBC packs transfused, delay from prescription to transfusion, immediate transfusions reactions) and patient disposition.

### Data collection

Three trained data collectors extracted data from patient charts and request forms from the period of January to June 2022. Request forms found to be duplicates or illegible / incomplete forms were removed. Data was recorded on paper forms and later entered into an Epi-Info database. The study principal investigator reviewed completed forms for completeness and accuracy. Informed consent was not required, given this review design.

### Data analysis

Data were entered into Epi Info version 7.2.5.0 for analysis. Qualitative variables were summarized using frequency distributions. Quantitative variables were summarized using mean and standard deviation or median and interquartile range. For the bivariate analysis, we used appropriate statistical tests, such as the Chi-square or Fisher exact test for categorical variables. The two groups of patients – transfused and not transfused- were analyzed according to certain demographic (age grouped, sex) and clinical characteristics (type of patient, death, length of stay in the ED, etc.). Similarly, delay from prescription to transfusion was analyzed with the degree of urgency of the RBC request, as well as mortality. The p-values were used to determine the statistical significance of the results, with an alpha threshold of 0.05.

### Ethical considerations

The study was approved by the Zanmi Lasante Institutional Review Board (Application number: ZL01042022).

## Results

**Fig. 1:**
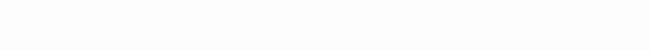
Selection of study cases

During the study period, 3,993 patients were managed at the ED of Mirebalais Teaching Hospital. Of these, 373 RBC request forms were completed. However, 64 of these forms were duplicates and were immediately excluded from the analysis. A further 47 RBC request forms were excluded for various reasons. [Figure 1] Finally, 262 RBC request forms, representing 84.78% of the initial forms, were retained for the analysis. Therefore, the demand for transfusion was 7.69% (307 out of 3993).

### Sociodemographic characteristics (*N* = 262)

Among the patients for whom pack RBCs were requested, there were slightly more male patients (145, 55.3%) than female patients (117, 44.7%) with a male-to-female ratio of 1.2. The median age of the patients was 43 [30–56] years, with a mean age of 44 years [range: 9–84]. The most represented age group was the 17–50 years old category, with 164 cases (62.12%). This was followed by the over-50 and under-17 age groups, comprising 94 cases (35.61%) and 5 cases (1.89%), respectively. The age of one adult patient was unknown, as an unknown individual brought the patient unconscious to the emergency department. This case was not considered when calculating the mean age. Patients seen at the ED of Mirebalais Teaching Hospital with conditions requiring blood transfusion came from nine out of the ten departments in the country, with the majority from the central department (110; 41.98%), followed by the West Department (100; 38.17%) and the Artibonite Department (34; 12.97%) (Table 1).

### Clinical characteristics (*N* = 262)

Most patients for whom a RBC request was made had non-traumatic conditions (medical and non-traumatic surgical cases), representing 75.38% of the patients. Trauma cases (fracture, blunt abdominal trauma, open abdominal trauma, and hemopneumothorax) accounted for 24.62% of the patients.

Apart from the indications “Probable Severe Anemia” (142; 54.19%) and “Preparation for the Operating Room” (103; 39.31%), the most common indications for transfusion were infections/sepsis, trauma, and cancer (39.31%, 36.64%, and 23.66%, respectively). Other less common indications included upper and lower gastrointestinal bleeding (7.25%), HIV-AIDS (6.87%), chronic kidney failure (6.1%), and hemorrhagic shock (3%). It should be noted that these indications are reported on request forms, and a single patient could have multiple indications for transfusion. Slightly over half of the patients (51.53%) had a hemoglobin level ≤ 7 g/dL, whereas approximately 20% had hemoglobin levels above 11 g/dL. The mean pre-transfusion hemoglobin level was 4.75 ± 1.68 g/dL (Table 1).

### RBCs request details (*N* = 262)

A total of 520 RBC pouches were obtained from the blood bank. In most cases (82.56%), two RBC bags were ordered per patient, regardless of the pre-transfusion hemoglobin level or underlying pathology. In 40.84% of cases, physicians deemed the transfusion request as an immediate life-threatening emergency by completing the blood request form, indicating that blood should be available immediately. In nearly half of the cases (48.47%), they regarded the request as a life-threatening emergency, meaning that blood should be available within the hour (Table 1).

**Table 1:** Demographic, clinical and pre-transfusion laboratory characteristics of patients for whom RBCs were requisitioned from the blood bank (*N* = 262)

Variables	Frequency (%)
Sex	
Female	117 (44.7%)
Male	145 (55.3%)
**Age**	
9 years - < 17 years	5 (1.91%)
17–50 years	162 (61.83%)
Over 50 years	94 (35.88%)
**Home department**	
Center	110 (41.98%)
West	100 (38.17%)
Artibonite	34 (12.97%)
Others (Frequency < 10)	18 (6.88%)
**Patient type**	
Non-trauma patients	197 (75.19%)
Trauma patients	65 (24.81%)
**Pre-transfusion hemoglobin level**	
≤ 7 g/dL	135 (51.53%)
7 - < 11 g/dL	74 (28.24%)
≥ 11 g/dL	53 (20.23%)
**Transfusion indications (Boolean field)**	
Probable severe anemia	142 (54.19%)
Preparation for the operating room	103 (39.31%)
Infection / Sepsis	96 (36.64%)
Trauma	62 (23.66%)
Cancer	57 (21.75%)
Gastrointestinal bleeding	19 (7.25%)
HIV /AIDS	18 (6.87%)
Chronic renal failure	16 (6.10%)
Others (Frequency ≤ 10)	59 (22.51%)
**Patient layout**	
Operating room (Orthopedics / General surgery)	91 (34.73%)
Hospital admission	69 (26.34%)
Patient’s discharge	65 (24.81%)
Emergency room deaths	26 (9.92%)
Others (Frequency < 10)	11 (4.20%)
**Quantity of 450 cc RBC packs required**	
One pack	22 (8.53%)
Two packs	213 (82.56%)
Three packs	21 (8.14%)
More than three packs	6 (2.29%)
**Degree of urgency of the request for RBC**	
Immediate vital emergency	107 (40.84%)
Vital emergency	127 (48.47%)
Relative urgency	21 (8.02%)
Not mentioned	7 (2.67%)

### Transfused patients (*N* = 57)

**Fig. 2:**
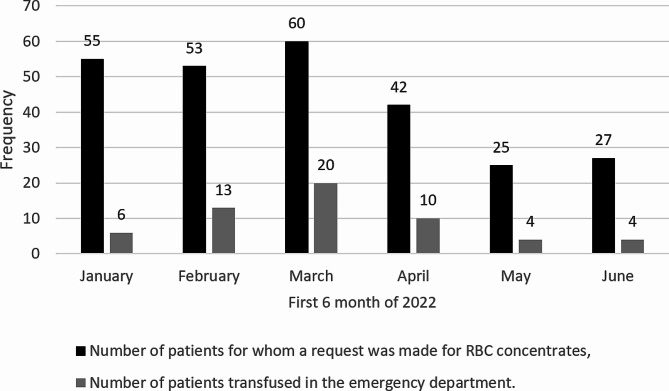
Breakdown by month of the number of patients for whom RBCs were requisitioned from the blood bank and the number of patients actually transfused in the ED of Mirebalais Teaching Hospital

Of the patients for whom an RBC request was made, 21.75% received a transfusion in the ED, with requests and transfused patients’ numbers varying considerably from month to month, with a higher peak in March (Figure 2) 79 RBC bags were transfused, accounting for 15.2% of the total number (520) of blood bags requested. Most patients received only one 450 cc pocket of RBCs in the emergency department. Unfortunately, one patient experienced an acute transfusion reaction (TRALI versus TACO), which proved to be fatal (Table 2). The median transfusion time delay was 2.37 days (IQR: 0.97–4.93 days). Less than a third of the patients (28.07%) were transfused within 24 h of the RBC request, while 22.82% of patients had to wait between five and more than ten days at the emergency department before receiving an RBC pack (Table 2).

**Table 2:** Delay from prescription to transfusion and acute transfusion reactions in transfused patients (*N* = 57)

Variables	Frequency (%)
Delay from prescription to transfusion	
Less than 24 h	16 (28.07%)
24 to < 72 h	15 (26.31%)
3 to < 5 days	13 (22.80%)
5 to 10 days	9 (15.80%)
More than 10 days	4 (7.02%)
**Quantity of 450 cc RBC packs transfused**	
One pack	41 (71.93%)
Two packs	12 (21.05%)
Three packs	3 (5.26%)
More than three packs	1 (1.75%)
**Acute transfusion reactions**	
No acute transfusion reactions	56 (98.25%)
TRALI vs. TACO	1 (1.75%)

Among patients with an immediate vital emergency demand, 22.43% (*n* = 24) were transfused, compared with 25.19% (*n* = 32) among those with a vital emergency demand. None of the patients with a relative emergency request (*n* = 21) were transfused; 91.23% of transfused patients had a hemoglobin level ≤ 7 g/dL (Table 3).

### Comparison of demographic and clinical characteristics between transfused and non-transfused patients

Several significant associations were observed between patient characteristics and the probability of receiving a blood transfusion during their visit to the ED among patients for whom a RBC request was made to the blood bank. Patients with trauma were less frequently transfused (*p* < 0.0001), while those with a hemoglobin level ≤ 7 g/dL (*p* < 0.0001) or patients discharged (*p* = 0.0171) were more likely to receive a transfusion. Similarly, the degree of urgency of the request for RBCs was significantly associated with the probability of transfusion (*p* = 0.0351). There appeared to be a correlation between length of stay in the ED and the probability of receiving a blood transfusion, with a significant increase in this probability as length of stay increased (*p* < 0.0001). In contrast, no significant association was found between patient age and probability of transfusion (*p* = 0.2786), despite a non-significant trend towards fewer transfusions in patients under 50 (Table 3).

Emergency room death do not appear to be significantly related to the delay from prescription to transfusion time, or to the degree of urgency of the request for RBC (Tables 4 and 5).

**Table 3:** Comparison of demographic and clinical characteristics between Transfused and Non-transfused patients

	Transfused Patient (*N* = 57)	Non-transfused Patient (*N* = 205)	OR [95% CI]	*P* value	Association test
**Age**					
≤ 50 years	33	134	0.71 [0.39–1.30]	0.2786	Chi-squared
Over 50 years	24	70	1		
**Sex**					
Female	39	78	3.53 [1.88–6.59]	< 0.0001	Chi-squared
Male	18	127	1		
**Patient type**					
Trauma patients	3	62	0.13 [0.03–0.39]	0.00002	Fisher exact
Non-trauma patients	54	143	1		
**Pre-transfusion hemoglobin level**					
≤ 7 g/dL	52	83	15.47 [5.92–40.38]	< 0.0001	Fisher exact
> 7 g/dL	5	122	1		
**Patient layout**					
Operating room (Orthopedics / General surgery)	14	77	1	0.0171	Fisher exact
Hospital admission	12	57	0.53 [0.26–1.10]		
Patient’s discharge	24	41	1.19 [0.66–2.17]		
Emergency room deaths	4	22	0.16 [0.05–0.49]		
Others	3	8	0.12 [0.03–0.44]		
**Degree of urgency of the request for RBC**					
Immediate vital emergency	24	83	1	0.0351	Fisher exact
Vital emergency	32	95	1.02 [0.59–1.77]		
Relative urgency	0	21	*Undefined*		
Not mentioned	1	6	0.025 [0.003–0.181]		
**Length of stay in ED from the time of RBC request**					
Less than 24 h	0	68	*Undefined*	< 0.0001	Fisher exact
24 to < 72 h	15	63	1		
3 to < 5 days	11	22	0.42 [0.19–0.91]		
5 to 10 days	13	28	0.51 [0.24–1.04]		
More than 10 days	18	23	0.74 [0.38–1.43]		

**Table 4:** Emergency room deaths according to delay from prescription to transfusion

Delay from prescription to transfusion	Emergency room deaths		P value	Association test
	**Yes (***N* = **4)**	**No (***N* = **53)**		
Less than 24 h	0	16	0.3000	Fisher exact
24 to < 72 h	1	14		
3 to < 5 days	1	12		
5 to 10 days	2	7		
More than 10 days	0	4		

**Table 5:** Degree of urgency of the request for RBC according to Delay from prescription to transfusion

Delay from prescription to transfusion	Immediate Vital emergency (*N* = 24	Vital Emergency (*N* = 32)	*P* value	Association test
Less than 24 h	7	9	0.0865	Fisher exact
24 to < 72 h	6	8		
3 to < 5 days	9	4		
5 to 10 days	2	7		
More than 10 days	0	4		

## Discussion

This article’ aims was to determine the proportion of patients requiring emergency transfusion, transfused patients’ characteristics, as well as the response rate and timeliness of blood product delivery for requests made. The main results were 7.69% prevalence of patients required RBC transfusion in the emergency department, the majority being non-traumatic and male. The blood bank was able to respond to only 15.2% of RBC requests, with a median time delay from prescription to transfusion of 2.37 days. The mean hemoglobin count before transfusion was 4.75 ± 1.68 g/dL. With regard to immediate complications associated with blood transfusions, our study recorded only one case of post-transfusion acute lung injury (TRALI) or post-transfusion circulatory overload (TACO).

The RBC transfusion prevalence in our study is similar to a study by Essola et al. (2015) in Gabon (6.7%) [8]; however, it is higher than that observed in Madagascar (3.32%; *N* = 51) [9] but lower than that of Rwanda (12.1%; *N* = 1116), which was almost twice as high as ours [14]. These differences may reflect variations in healthcare infrastructure, disease epidemiology, and access to healthcare services across different regions.

The mean age of 44 years in our study was much lower than in high-income countries (around 70 years) [7, 15, 16] but similar to Essola et al. (2015) and Raveloson et al. (2012) in Gabon and Madagascar, respectively, 40.8 ± 16 years [9] and 48.5 years [8]. However, it is essential to note that non-traumatic pediatric cases were not included in our study, preventing us from assessing the burden of pediatric patients requiring RBC transfusion at the Mirebalais Teaching Hospital’s ED. The mean pre-transfusion hemoglobin was also remarkably low compared to other studies reviewed [9]. ^,^ [7, 16].

Our study found that only 15.2% of RBC requests were met by the blood bank, compared to 78.7% in the Indian study by Christopher et al. (2020) [17]. The median delay from prescription to transfusion was over two days versus about 6 h in the African studies by Essola et al. and Raveloson et al. [8]. ^,^ [9] Forty-one of the 65 patients discharged were not transfused, 24 had a hemoglobin level below 8 g/dL. However, these non-transfused patients were placed on injectable iron, which raised their hemoglobin levels a little before discharge. The present study reveals severely constrained blood product availability with prolonged procurement delays at the ED of Mirebalais Teaching Hospital. These findings underscore major challenges in accessing blood products in this emergency care department. This observation aligns with the established data on the limited availability of blood products in low-income countries. These countries typically have only 5.0 blood donations per 1000 inhabitants [3]. The shortage of blood products appears to be even more severe in Haiti, where the number of blood units collected nationwide in 2014 was 28,867, equivalent to 0.27% of the Haitian population that year (10.41 million inhabitants) [18]. 

Additionally, the analysis of demographic and clinical characteristics provided valuable insights into factors influencing the probability of receiving a blood transfusion. Trauma patients were less frequently transfused, while patients with lower hemoglobin levels, those discharged from the hospital, and those with longer lengths of stay in the ED were more likely to receive transfusions. The predominance of female transfusions in our study does not correlate with other African and European studies in our literature review [6, 9, 14, 16]. Further investigation is warranted to elucidate the complex interplay of these factors and their impact on transfusion practices, thus facilitating the development of targeted interventions to ensure equitable and effective healthcare delivery.

Emergency room deaths were not found to be significantly related to the delay from prescription to transfusion time or to the degree of urgency of the request for RBC. This suggests that while delays in transfusion may pose risks to patient outcomes, other factors may also contribute to mortality in the ED setting, warranting further investigation.

This study had some limitations. First, it is an retrospective study; therefore, we relied on data available in the medical records to collect the necessary information. Additionally, this study was conducted at a single hospital, which limits the generalizability of the results to other emergency care settings. Another limitation is that we examined only 6 months of data, which may represent only a part of the year. In addition, this study focused only on RBC requests and did not examine other labile blood products because the Mirebalais Teaching Hospital blood bank only stocks RBCs. Caution should be taken with the use of associations resulting from bivariate analyses because of the small size of transfused patients’ group (*N* = 57). Results, sensitive to random variations, may be less generalizable, requiring confirmation with larger samples for a more robust interpretation.

Despite these limitations, this study provides valuable information regarding the prevalence of transfusions in EDs. This serves as a solid foundation for future research to improve transfusion practices in emergency services and address challenges related to the availability of blood products in low-income countries.

## Conclusion

This study’s results revealed that a relatively significant proportion of patients required RBC transfusions, highlighting the importance of this need for emergency services. We also observed a relatively low response capacity to this demand and a high response time, indicating difficulty accessing blood products at Mirebalais Teaching Hospital. Therefore, it is essential to conduct further studies to assess the appropriateness of transfusion requests to optimize the use of RBCs and ensure an adequate response from patients who genuinely need them, thus rationalizing transfusion practices in emergency services.

The limited availability of blood products in low-income countries remains a significant challenge, and measures must be taken to improve the supply and requisition-delivery processes to meet the growing needs of emergency services.

## Data Availability

No datasets were generated or analysed during the current study.
